# Evaluation of White Matter Integrity Utilizing the DELPHI (TMS-EEG) System

**DOI:** 10.3389/fnins.2020.589107

**Published:** 2020-12-21

**Authors:** Ofri Levy-Lamdan, Noa Zifman, Efrat Sasson, Shai Efrati, Dallas C. Hack, David Tanne, Iftach Dolev, Hilla Fogel

**Affiliations:** ^1^QuantalX Neuroscience, Beer-Yaacov, Israel; ^2^Sagol Center for Hyperbaric Medicine and Research, Shamir Medical Center, Zerifin, Israel; ^3^Sackler School of Medicine and Sagol School of Neuroscience, Tel-Aviv University, Tel-Aviv, Israel; ^4^Department of Physical Medicine and Rehabilitation, Virginia Commonwealth University, Richmond, VA, United States; ^5^Stroke and Cognition Institute, Rambam Healthcare Campus, Haifa, Israel

**Keywords:** brain, white matter, DELPHI, connectivity, imaging, network, stroke, TBI

## Abstract

**Objective:**

The aim of this study was to evaluate brain white matter (WM) fibers connectivity damage in stroke and traumatic brain injury (TBI) subjects by direct electrophysiological imaging (DELPHI) that analyzes transcranial magnetic stimulation (TMS)-evoked potentials (TEPs).

**Methods:**

The study included 123 participants, out of which 53 subjects with WM-related pathologies (39 stroke, 14 TBI) and 70 healthy age-related controls. All subjects underwent DELPHI brain network evaluations of TMS-electroencephalogram (EEG)-evoked potentials and diffusion tensor imaging (DTI) scans for quantification of WM microstructure fractional anisotropy (FA).

**Results:**

DELPHI output measures show a significant difference between the healthy and stroke/TBI groups. A multidimensional approach was able to classify healthy from unhealthy with a balanced accuracy of 0.81 ± 0.02 and area under the curve (AUC) of 0.88 ± 0.01. Moreover, a multivariant regression model of DELPHI output measures achieved prediction of WM microstructure changes measured by FA with the highest correlations observed for fibers proximal to the stimulation area, such as frontal corpus callosum (*r* = 0.7 ± 0.02), anterior internal capsule (*r* = 0.7 ± 0.02), and fronto-occipital fasciculus (*r* = 0.65 ± 0.03).

**Conclusion:**

These results indicate that features of TMS-evoked response are correlated to WM microstructure changes observed in pathological conditions, such as stroke and TBI, and that a multidimensional approach combining these features in supervised learning methods serves as a strong indicator for abnormalities and changes in WM integrity.

## Introduction

The disruption of normal patterns of structural brain connectivity is believed to play a central role in the pathophysiology of many neurological and psychiatric disorders, such as dementia, movement disorders, stroke, traumatic brain injury (TBI), etc. White matter (WM) pathways consist of myelinated axonal structures that constitute the connectivity between different brain regions. Axonal injury and degeneration may occur even in the absence of tissue disruption. Therefore, patients may experience significant impairment despite the absence of abnormal findings on conventional CT or MRI. Moreover, focal imaging abnormalities that can be detected using CT and MRI are poor predictors of outcome (Niogi and Mukherjee, 2010; Adalbert and Coleman, 2013; Wang et al., 2016; Salvadores et al., 2017).

Axonal injury is a key determinant of clinical outcome in cases of brain injury and has been shown to be an important factor determining long-term motor, cognitive, and neuropsychiatric disability following brain injury (Mac Donald et al., 2007; Di Paola et al., 2010; Moura et al., 2019). Diagnostic tests that can discriminate significant axonal injury and degeneration are needed in order to accurately assess injury severity and effectively determine treatment and follow-up pathway.

WM pathways determined through diffusion tensor imaging (DTI) are traditionally considered to be the biophysical representation of axonal bundles and their myelin sheets. DTI streamlines between cortical and subcortical gray matter (GM) regions of interest (ROIs) can be used as a measure of the magnitude and strength of connection between ROIs (Basser et al., 1994; Le Bihan et al., 2001). Analyzing changes in brain connectivity using DTI tractography is widely used to evaluate axonal injury that is a hallmark of stroke and TBI (Arfanakis et al., 2002; Lo et al., 2010), in which WM tracks can be injured directly or indirectly through Wallerian degeneration (the anterograde distal degeneration of injured axons accompanied by demyelination) (Chen et al., 2017; Moura et al., 2019). DTI metrics are used to address specific or diffused WM damage that is frequently affected by stroke lesions in order to describe stroke focality and severity and predict rehabilitation potential (Puig et al., 2013; Puig et al., 2020). In TBI patients, a consistent reduction in fractional anisotropy (FA) has been typically found in areas affected by traumatic axonal injury (TAI). These regions include the subcortical WM of the frontal and temporal regions, the splenium of the corpus callosum, the posterior limb of the internal capsule, and the cerebral peduncles (Nakayama et al., 2006; Benson et al., 2007; Xu et al., 2007; Greenberg et al., 2008).

Combining transcranial magnetic stimulation (TMS) with electroencephalogram (EEG) is extensively used to study and assess cerebral reactivity and connectivity (Rossini et al., 2015; Ferreri et al., 2017; Tremblay et al., 2019). TMS is a non-invasive brain stimulation method that allows the study of human cortical function *in vivo* (Ilmoniemi et al., 1999; Hallett, 2007). TMS enables the modulation and exploration of brain functional status. Studies integrating TMS with EEG (TMS-EEG) have shown that TMS produces waves of activity that reverberate throughout the cortex and that are reproducible and reliable (Casarotto et al., 2010; Farzan et al., 2010; Kerwin et al., 2018), thus providing direct information about cortical excitability and connectivity with excellent temporal resolution (Ilmoniemi et al., 1997; Shafi et al., 2012; Chung et al., 2015; Pennisi et al., 2016; Bordet et al., 2017). TMS-EEG has been used to causally probe the dynamic effective connectivity of human brain networks (Rogasch and Fitzgerald, 2013; Ferreri et al., 2014; Kugiumtzis and Kimiskidis, 2015).

Direct electrophysiological imaging (DELPHI) is a new clinical methodology for evaluating fundamental physiological properties of brain network function by automatically combining TMS stimulation and EEG monitoring (Zifman et al., 2019). The DELPHI software algorithm extracts direct stimulation-related properties of brain network function, characterizing a profile of brain functional pathophysiology including properties of network integrity and plasticity.

Several studies have explored the correlation of TMS-evoked response to WM fibers and GM changes, indicating that this method may be useful for probing neurological disease and conditions that manifest changes in white or GM ROI’s (Kirton et al., 2010; Lanza et al., 2013; Pennisi et al., 2015).

The current study tests DELPHI technology and output parameters as a clinical tool for evaluation of specific neuronal tracks connectivity and displays its strong correlation to structural changes in WM fibers. The study population includes stroke and TBI patients that represent the population with connectivity disruption caused predominantly by WM changes and healthy controls.

## Materials and Methods

### Clinical Data Collection and Analysis

The study was carried out in accordance with the recommendation of the Shamir Medical center review board. The protocol was approved by the local institutional “Ethical Committee” as a retrospective study of data. All participants underwent the exact same MRI scan and DELPHI evaluation protocols.

### Study Population

The study focused on two main groups: (a) healthy controls (HC) and (b) subjects diagnosed with WM injury-related conditions either (b1) ischemic stroke (cortical and subcortical) or (b2) TBI. All stroke/TBI subjects were considered to be at post-sub-acute stage of the injury, post-injury rehabilitation, and discharged from hospitalization. All subjects included in the study had updated documented medical history, demographics, clinical MRI evaluation, DTI scan, and DELPHI (TMS-EEG) scan performed within 2 weeks of each other. Healthy subject group inclusion criteria (a) were as follows: (1) age over 18 years, (2) no neurological or psychiatric disorder documented in medical history or self-report, (3) absence of any significant abnormal findings in clinical MRI evaluation, such as brain tumors, subdural hematoma, and other brain structural lesions related to diagnosed brain disease other than common age-related changes, and 4. no psychoactive or other brain directed medications. Stroke subject group (b1) inclusion criteria were as follows: (1) age over 18 years, (2) history of ischemic stroke over 6 months and less than 5 years prior to MRI, DTI, and TMS-EEG scans (post-sub-acute stage and rehabilitation), (3) injury was detectible by clinical MRI scan, and (4) no other psychiatric or neurological comorbidities. TBI subject group (b2) inclusion criteria were as follows: (1) age over 18 years, (2) history of TBI over 6 months and less than 5 years prior to MRI, DTI, and TMS-EEG scans (post-sub-acute stage and rehabilitation), (3) injury was detectible by clinical MRI scan, and 4. no other psychiatric or neurological comorbidities. Demographic information of the study groups is listed in Tables 1, 2. Due to the age differences of the TBI and stroke populations, the healthy population was divided into age-matched groups to serve as a valid control group for each of the WM conditions populations.

**TABLE 1:** **(A)** Demographics of stroke and age-matched HC subjects. Age distribution was not significantly different (*p* > 0.05). **(B)** Overview of general injury location detected by clinical MRI.

(A)		

	Stroke	Healthy
N	39	41
Age (Mean ± SD)	66.6 (±7.4)	63.8 (±8.1)
Male	28	32
Female	11	9
Left hand-dominance	5	3
Time from injury (Mean ± SD, in years)	2.4 (±2)	

**(B)**				

**Area**	***n* of patients**			
		**Left**	**Right**	**Both**
Cortical	2	1	1	–
Cortical + subcortical	9	4	2	3
Subcortical/Deep	18	9	5	4
Cerebellum/Brain stem	10	3	3	4
Total	39			

**TABLE 2:** **(A)** Demographics of TBI and HC subjects. Age distribution was not significantly different (*p* > 0.05). **(B)** Overview of general injury location detected by clinical MRI.

(A)		

	TBI	Healthy
*N*	14	29
Age (Mean ± SD)	32.5 (±5.9)	34.1(±5.6)
Male	11	17
Female	3	12
Left hand-dominance	2	2
Time from injury (Mean ± SD, in years)	3.6 (±1.4)	

**(B)**				

**Area**	***n* of patients**			
		**Left**	**Right**	**Both**

Cortical	2	1	0	1
Cortical + subcortical	6	1	0	5
Subcortical/Deep	3	1	0	2
Cerebellum/Brain Stem	3	0	0	3
Total	14			

### DTI Imaging

All subjects underwent brain MRI scan and DELPHI evaluation not more than 2 weeks apart. Imaging was performed with a 3 Tesla system (MAGNETOM Skyra; Siemens Healthineers, Erlangen, Germany) with 20 channels receiver head coil. Thirty diffusion-weighted images were scanned with different gradient directions (*b* = 1,000) and one volume without diffusion weighting, with the following parameters: *TR* = 10,300 ms, *TE* = 89 ms, voxel size = 1.8 × 1.8 mm, matrix = 128 × 128, no. of slices = 63, slice thickness = 2.2 mm.

#### DTI Analysis

DTI analysis was performed on the FA map calculated by Siemens post-processing software. For each subject, the WM atlas (ICBM-MORI white matter atlas (Mori et al., 2008) was registered to the FA map using SPM (version 12; The Wellcome Centre for Human Neuroimaging, UCL Queen Square Institute of Neurology, London, United Kingdom) and manually validated. Mean values for FA higher than 0.2 were calculated in WM regions according to the atlas (Wakana et al., 2004, 2007; Hua et al., 2008).

### TMS-EEG

TMS was performed with a MagPro R30 stimulator (MagVenture, Denmark) and an MCF-B65-HO figure-8 Coil (MagVenture, Denmark). 32-Channel EEG data were obtained using TMS compatible BrainAmp DC amplifier (5 kHz sampling rate, ± 16.384 mV measurement range, analog low pass filter 1 kHz; Brain Products GmbH, Germany). These were connected to the waveguard^TM^ EEG cap (ANT Neuro, Netherland) with Ag-AgCl electrodes. The reference and ground electrodes were affixed to the ear lobes. EEG data were recorded using a BrainVision Recorder software (Brain Products GmbH, Germany).

### Experimental Procedure

TMS coil was positioned over the left cortical motor (M1) region, at 45° toward the contralateral forehead according to the guidelines (Rossini et al., 2015). Single pulse (<0.3 Hz frequency and 1 Hz inhibitory frequency) stimulation was performed at 80% from rest motor threshold (RMT) intensity. Data acquisition, preprocessing, and cleaning of the TMS-evoked response include rejection of bad channels and epochs containing large artifacts, followed by bandpass FIR filter (0.5–45 Hz) as detailed by Zifman et al. (2019). A thin (0.5 mm) foam pad was attached to the TMS coil to minimize electrode movement and bone-conducted auditory artifact. Participants were instructed to keep their eyes closed throughout the examination to reduce ocular artifacts. The operator of the system conversed with subjects between the short stimulation protocol blocks in order to avoid drowsiness. Electrodes data were grouped to regional recording hotspots for analysis and statistical purposes: frontal: F3, F5—ipsilateral and F4, F6—contralateral to stimulation; parietal: C3, C5, CP1—ipsilateral and C4, C6, CP2—contralateral to stimulation; temporal CP5, CP3, CF5—ipsilateral and CP6, CP4, FC6—contralateral to stimulation; and occipital cortex: O1, PO3—ipsilateral and O2, PO4—contralateral to stimulation.

### DELPHI Physiological Network Profile Analysis Parameters

DELPHI analyzes the regional and network TMS-evoked potential (TEP) measured as EEG patterns of single and history-dependent events as described by Zifman et al. (2019). TEP response refers to 20–300 ms after TMS stimulation and was described by four parameters: (1) the slope of early response, between 60 and 100 ms, referred to as early phase deflection (EPD); (2) the slope of late response, between 100 and 180 ms, referred to as late phase deflection (LPD); (3) waveform adherence (WFA) refers to the entire TEP response adherence to mean healthy age-related signal collected from previous studies; and (4) the normalized difference ratio between mean field potential of single pulse response (MFP_single_) and mean field potential of inhibitory frequency of stimulation (MFP_i_), indicating network short-term plasticity (STP) calculated as (MFP_single_ − MFP_i_)/(MFP_single_ + MFP_i_). All data processing and feature extraction were performed automatically by the DELPHI software algorithm.

### Classification

Linear Support Vector Machine (SVM) classifier, with 50 permutations of stratified 5-fold cross-validation and class prior probabilities set to reflect realistic balanced proportions, was used for classification of population with WM-related conditions (TBI and stroke) and age-matched healthy population by DELPHI output measures as features vector. Linear SVMs are known as stable classifiers with low complexity that enables accommodation to outliers and are relatively insensitive to overtraining and curse-of-dimensionality (Lotte et al., 2007, 2018; Li et al., 2014). In order to achieve maximal signal-to-noise ratio (SNR) and decrease the feature vector length, only electrode hotspots that are closest to the stimulation site were used in the model construction (left/right temporal and parietal hotspots) as they are known to be the most reliable and are traditionally used in TMS-EEG studies (Lioumis et al., 2009; Ilmoniemi and Kicić, 2010). Feature vector included all four DELPHI output measures. After training the model on the train data set, the model’s classification performance was assessed on the test data set by extracting balanced accuracy, sensitivity (true positive rate), specificity (true negative rate), mean, and standard deviation (STDV) values over all permutations. Receiver Operating Characteristic (ROC) curve was constructed by plotting the mean true positive rate (sensitivity) against the mean false positive rate (1 - specificity) with the various cut-off thresholds.

### Regression

WM ROIs FA was calculated for major fiber tracks, and a multivariate linear regression model was created to predict ROI’s FA high-dimensional data using DELPHI features vector (described above) in all study populations sets pooled together (healthy, stroke, and TBI populations) (Healy, 1980). The model was used with 50 permutations of 5-fold cross-validation, and prediction performance was assessed by Pearson’s correlation coefficients and root-mean-square error (RMSE), mean, and STDV over all permutations.

### Statistical Analysis

Statistical data analysis to account for the differences between the groups was performed by two samples *t*-test, followed by the Bonferroni–Holm method for multiple comparisons correction. Corrected *p*-values are depicted in text, and the significance of the results following multiple comparisons correction is depicted by asterisks: ^∗^*p* < 0.05, ^∗∗^*p* < 0.01, ^∗∗∗^*p* < 0.001, ns—non-significant. All data statistical analysis was made using MATLAB (R2020a; The Mathworks Inc., MA, United States).

## Results

The current study focuses on TMS-EEG neurophysiological insight into brain network function and connectivity by correlating TMS-evoked EEG response to WM fibers integrity. For this reason, a heterogenic group of subjects who sustained structural connectivity injuries in potentially different areas were randomly selected. The study groups include post-rehabilitation, ischemic stroke subjects with lesions of different sizes, post-rehabilitation TBI subjects, and healthy subjects. Tables 1A,B, 2A,B summarize age, gender, hand dominance, and general brain region of injury.

### Evaluation of WM Changes in Stroke/TBI

ROI-based comparison of WM tracks FA displayed, as expected, significantly reduced mean FA and elevated STDV for the majority of measured WM fibers both in the stroke group compared with HC and in the TBI group compared with age-matched HC (Table 3). The significant reduction of FA values in a wide range of ROIs is consistent with the heterogenicity of study population injuries and variability of fiber damage locations and sizes in both stroke and TBI.

**TABLE 3:** ROI-based FA analysis of stroke and TBI compared with age-matched HC.

ROI		HC		Stroke		*p*-value (corrected)		HC		TBI		*P*-value (corrected)	
		Mean	STD	Mean	STD			Mean	STD	Mean	STD		
Middle cerebellar peduncle		0.32	0.11	0.32	0.09	0.97	ns	0.28	0.11	0.32	0.1	0.28	ns
Pontine crossing tract		0.41	0.03	0.35	0.04	0	***	0.42	0.04	0.34	0.05	0	***
Corpus callosum	Genu	0.52	0.04	0.4	0.06	0	***	0.57	0.03	0.39	0.09	0	***
	Body	0.56	0.03	0.44	0.07	0	***	0.59	0.02	0.36	0.1	0	***
	Splenium	0.6	0.04	0.49	0.09	0	***	0.62	0.03	0.39	0.11	0	***
Corticospinal tract	Left	0.43	0.04	0.38	0.05	0	***	0.41	0.04	0.37	0.06	0.05	*
	Right	0.43	0.04	0.38	0.05	0	***	0.42	0.04	0.38	0.06	0.05	*
Medial lemniscus	Left	0.43	0.04	0.37	0.05	0	***	0.42	0.04	0.37	0.06	0.03	*
	Right	0.43	0.05	0.37	0.05	0	***	0.42	0.05	0.36	0.06	0.01	*
Inferior Cerebellar peduncle	Left	0.4	0.04	0.36	0.05	0	***	0.4	0.04	0.36	0.05	0.04	*
	Right	0.41	0.04	0.36	0.05	0	***	0.4	0.03	0.36	0.06	0.03	*
Superior Cerebellar peduncle	Left	0.49	0.03	0.41	0.06	0	***	0.48	0.04	0.38	0.08	0	***
	Right	0.5	0.03	0.41	0.06	0	***	0.48	0.04	0.38	0.07	0	***
Anterior limb of internal capsule	Left	0.51	0.04	0.4	0.07	0	***	0.53	0.02	0.41	0.07	0	***
	Right	0.53	0.04	0.44	0.06	0	***	0.54	0.03	0.46	0.08	0	***
Posterior limb of internal capsule	Left	0.57	0.03	0.47	0.09	0	***	0.58	0.02	0.48	0.12	0	**
	Right	0.57	0.03	0.5	0.07	0	***	0.59	0.03	0.51	0.1	0	**
Retrolenticular internal capsule	Left	0.54	0.03	0.46	0.08	0	***	0.55	0.02	0.44	0.09	0	***
	Right	0.53	0.03	0.47	0.06	0	***	0.54	0.02	0.47	0.07	0	***
Anterior corona radiata	Left	0.43	0.03	0.35	0.05	0	***	0.46	0.02	0.39	0.06	0	***
	Right	0.44	0.04	0.36	0.05	0	***	0.47	0.03	0.4	0.05	0	***
Superior corona radiata	Left	0.47	0.03	0.38	0.09	0	***	0.48	0.03	0.43	0.06	0.02	*
	Right	0.46	0.03	0.4	0.06	0	***	0.47	0.03	0.43	0.06	0.02	*
Posterior corona radiata	Left	0.46	0.03	0.37	0.08	0	***	0.46	0.03	0.41	0.06	0	**
	Right	0.46	0.04	0.4	0.05	0	***	0.47	0.03	0.41	0.05	0	***
Thalamic radiation	Left	0.53	0.04	0.4	0.11	0	***	0.55	0.03	0.42	0.12	0	***
	Right	0.54	0.06	0.46	0.08	0	***	0.56	0.03	0.43	0.1	0	***
Sagittal stratum	Left	0.5	0.02	0.4	0.08	0	***	0.52	0.03	0.39	0.11	0	***
	Right	0.54	0.03	0.46	0.07	0	***	0.55	0.02	0.46	0.07	0	***
External capsule	Left	0.48	0.02	0.38	0.07	0	***	0.49	0.02	0.4	0.07	0	***
	Right	0.47	0.03	0.4	0.06	0	***	0.49	0.02	0.41	0.06	0	***
Cingulum	Left	0.55	0.04	0.43	0.08	0	***	0.54	0.04	0.35	0.11	0	***
Cingulategyrus													
	Right	0.51	0.04	0.41	0.07	0	***	0.51	0.03	0.34	0.1	0	***
Cingulum	Left	0.49	0.04	0.41	0.07	0	***	0.49	0.03	0.35	0.09	0	***
Hippocampus													
	Right	0.46	0.05	0.41	0.07	0	***	0.47	0.03	0.37	0.07	0	***
Fornix	Left	0.49	0.03	0.39	0.07	0	***	0.52	0.03	0.36	0.09	0	***
	Right	0.49	0.04	0.41	0.05	0	***	0.52	0.04	0.39	0.09	0	***
	Column and body	0.42	0.03	0.35	0.04	0	***	0.43	0.03	0.35	0.05	0	***
Superior longitudinal fasciculus	Left	0.5	0.03	0.37	0.11	0	***	0.52	0.03	0.38	0.09	0	***
	Right	0.48	0.04	0.39	0.09	0	***	0.51	0.03	0.38	0.07	0	***
Superior fronto occipital fasciculus	Left	0.5	0.06	0.33	0.09	0	***	0.54	0.03	0.38	0.1	0	***
	Right	0.5	0.07	0.36	0.08	0	***	0.54	0.03	0.41	0.11	0	***
Uncinate fasciculus	Left	0.49	0.05	0.42	0.11	0	***	0.5	0.05	0.37	0.1	0	***
	Right	0.54	0.04	0.46	0.06	0	***	0.53	0.04	0.42	0.07	0	***
Tapetum	Left	0.53	0.12	0.31	0.14	0	***	0.6	0.05	0.32	0.14	0	***
	Right	0.38	0.1	0.31	0.11	0	**	0.49	0.11	0.32	0.15	0	**

***p < 0.001; **p < 0.01; *p < 0.05; ns, non-significant.

### DELPHI Network Functional Analysis of Healthy vs. Stroke/TBI

**FIGURE 1:**
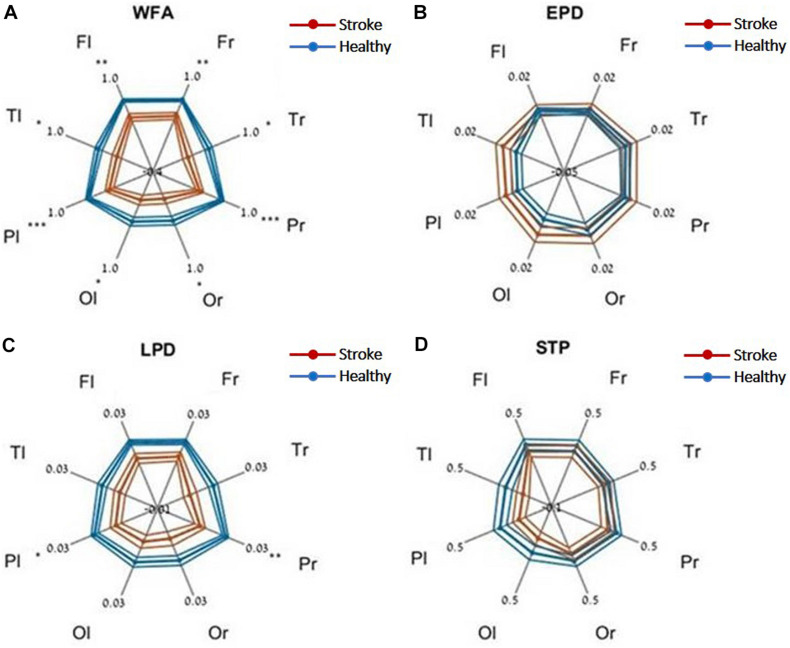
DELPHI output measures of HC vs. the stroke group at different recording sites. **(A)** WFA, **(B)** EPD, **(C)** LPD, **(D)** STP. Fl, frontal left; Fr, frontal right; Pl, parietal left; Pr, parietal right; Tl, temporal left; Tr, temporal right; Ol, occipital left; Or, occipital right. Mean values (thick lines) and SE (narrow lines) are depicted. Significant differences between populations are indicated by asterisks.

**FIGURE 2:**
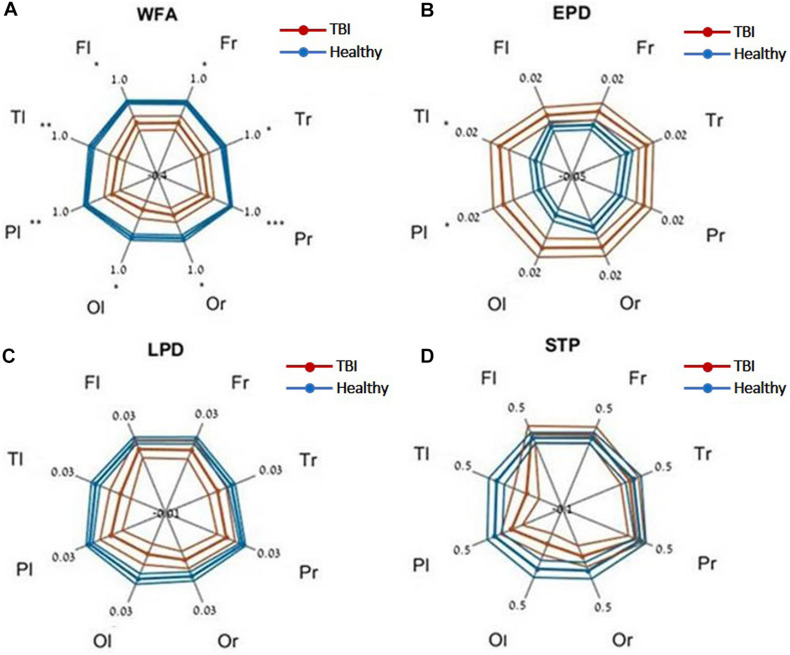
DELPHI output measures of HC vs. the TBI group at different recording sites. **(A)** WFA, **(B)** EPD, **(C)** LPD, **(D)** STP. Fl, frontal left; Fr, frontal right; Pl, parietal left; Pr, parietal right; Tl, temporal left; Tr, temporal right; Ol, occipital left; Or, occipital right. Mean values (thick lines) and SE (narrow lines) are depicted. Significant differences between populations are indicated by asterisks.

**FIGURE 3:**
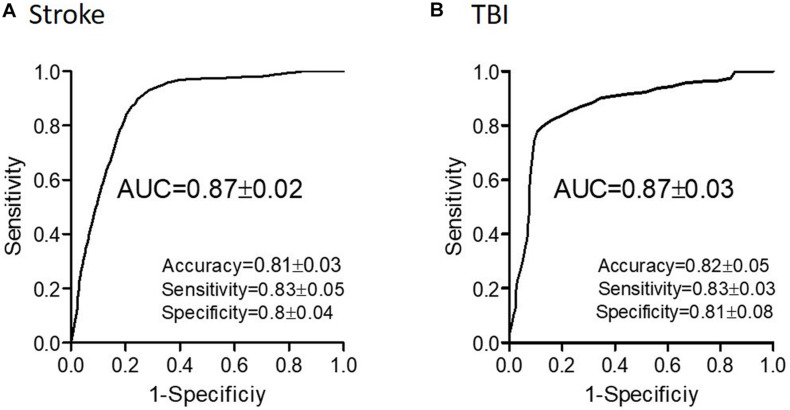
Classification performance of WM-related conditions using DELPHI output measures. Classification of stroke **(A)** and of TBI **(B)** and age-matched HC using DELPHI output measures as feature vector. Classification performance is depicted by the ROC curve and standard statistical measures, and balanced accuracy (accuracy), sensitivity, specificity, and AUC values are detailed in the figure.

Evaluating network function using DELPHI output parameters revealed a significant difference between the study groups. Significant differences were observed between stroke and age-matched HC for WFA in all regional hotspots (Figure 1A). EPD, which describes the early phase of response, did not display significant differences between the groups (Figure 1B); however, LPD displayed a significant difference for parietal hotspots (Figure 1C). STP index did not display significant differences (Figure 1D). DELPHI evaluation of TBI population compared with age-matched HC revealed a significant decrease in WFA in all recording hotspots (Figure 2A). EPD displayed significant differences in the left temporal and parietal areas, proximal to the stimulation area (Figure 2B). LPD and STP did not display any significant change (Figures 2C,D). A multidimensional approach combining DELPHI output measures using a linear SVM classifier is depicted in the model’s cross-validated classification performance. DELPHI TMS-EEG output parameters were able to differentiate HC from stroke subjects, at balanced accuracy, sensitivity, and specificity rates of 0.81 ± 0.03, 0.83 ± 0.05, and 0.8 ± 0.04, respectively. ROC area under the curve (AUC) was 0.87 ± 0.02 (Figure 3A). For TBI and HC, AUC was 0.87 ± 0.03, and classification balanced accuracy, sensitivity, and specificity rates were 0.82 ± 0.05, 0.83 ± 0.03, and 0.81 ± 0.08, respectively (Figure 3B). Pooling together all WM injury subjects (stroke and TBI) displayed a classification balanced accuracy of 0.81 ± 0.02, sensitivity rate of 0.82 ± 0.03, specificity of 0.8 ± 0.03, and AUC of 0.88 ± 0.01.

### Prediction of WM Abnormalities Using DELPHI

**FIGURE 4:**
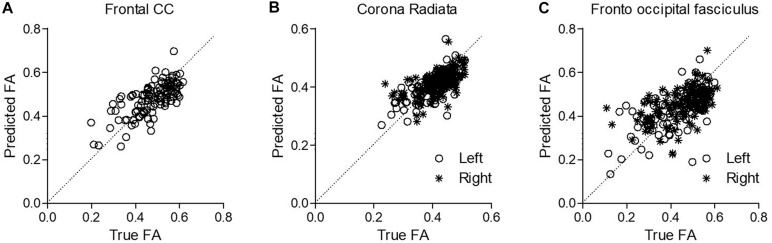
Prediction of WM abnormalities using DELPHI multivariate linear regression model on the pooled study groups collected data. Predicted FA values (*Y*-axis) were plotted against the true FA measured values (*X*-axis) for exemplary fibers. **(A)** Frontal commissural fiber—corpus callosum. **(B)** Left projection fiber—anterior corona radiata. **(C)** Left association fiber—superior fronto-occipital fasciculus.

Next, we evaluated the capacity of DELPHI (TMS-EEG) output measures to predict WM-related connectivity abnormalities captured by DTI–FA values, using a multivariate linear regression model. All study groups were pooled together (HC, stroke, and TBI), and WM ROIs FA was calculated for major fiber tracks. Cross-validated results of the multivariate model exhibit high correlation between DELPHI output parameters and DTI–FA values of WM fibers. Highest correlations were observed for left and frontal tracts (Table 4), most significantly for frontal commissural fiber—corpus callosum (*r* = 0.7 ± 0.02, RMSE = 0.07) (Figure 4A), left projection fiber—anterior corona radiata (*r* = 0.66 ± 0.02, RMSE = 0.04) (Figure 4B), and left association fiber—superior fronto-occipital fasciculus (*r* = 0.65 ± 0.02, RMSE = 0.09) (Figure 4C).

**TABLE 4:** WM ROIs FA values prediction by multivariate linear regression model of DELPHI output measures.

ROI		*R*		RMSE	
		Mean	STDV	Mean	STDV
Corpus callosum	Genu	0.7	0.02	0.07	0.002
	Body	0.58	0.03	0.08	0.003
	Splenium	0.52	0.03	0.09	0.003
Corticospinal tract	Left	0.34	0.04	0.05	0.002
	Right	0.37	0.04	0.05	0.001
Medial lemniscus	Left	0.35	0.04	0.05	0.002
	Right	0.36	0.04	0.05	0.002
Cerebellar peduncle Inferior	Left	0.4	0.03	0.05	0.001
	Right	0.37	0.04	0.05	0.001
Cerebellar peduncle Superior	Left	0.5	0.03	0.06	0.002
	Right	0.53	0.03	0.06	0.002
Anterior limb of internal capsule	Left	0.7	0.02	0.06	0.001
	Right	0.44	0.03	0.06	0.002
Posterior limb of internal capsule	Left	0.55	0.04	0.07	0.003
	Right	0.37	0.04	0.06	0.002
Retrolenticular part of internal capsule	Left	0.57	0.03	0.06	0.002
	Right	0.48	0.03	0.05	0.002
Anterior corona radiata	Left	0.66	0.02	0.04	0.001
	Right	0.56	0.02	0.05	0.001
Superior_corona_radiata	Left	0.55	0.04	0.06	0.003
	Right	0.29	0.05	0.06	0.002
Posterior corona radiata	Left	0.56	0.03	0.06	0.002
	Right	0.37	0.04	0.05	0.002
Thalamic radiation	Left	0.61	0.03	0.08	0.003
	Right	0.46	0.04	0.08	0.003
Sagittal stratum	Left	0.59	0.02	0.07	0.002
	Right	0.37	0.04	0.06	0.002
External capsule	Left	0.57	0.03	0.06	0.002
	Right	0.42	0.03	0.05	0.001
Cingulum cingulate gyrus	Left	0.5	0.03	0.08	0.003
	Right	0.51	0.03	0.07	0.002
Cingulum hippocampus	Left	0.48	0.03	0.07	0.002
	Right	0.19	0.05	0.07	0.002
Fornix	Left	0.6	0.03	0.07	0.002
	Right	0.4	0.03	0.07	0.002
	Column and body	0.64	0.02	0.04	0.001
Superior longitudinal fasciculus	Left	0.62	0.03	0.08	0.003
	Right	0.52	0.03	0.07	0.002
Superior fronto occipital fasciculus	Left	0.65	0.02	0.09	0.003
	Right	0.47	0.03	0.09	0.003
Uncinate fasciculus	Left	0.35	0.04	0.09	0.003
	Right	0.28	0.04	0.07	0.002
Tapetum	Left	0.48	0.03	0.15	0.005
	Right	0.32	0.04	0.13	0.005

*Models performance is depicted by Pearson’s correlation coefficient (R) and root-mean-square error (RMSE).*

## Discussion

In the present study, we evaluated the performance of DELPHI output parameters as biomarkers for structural and functional brain connectivity abnormalities, captured by WM tracks integrity measures, in stroke and TBI patients. Study results demonstrate the changes between HC and TBI or stroke populations for DELPHI output measures. The most significant difference of both stroke and TBI patients from healthy age-matched controls was demonstrated by WFA that represents the general waveform of single pulse TEP response. Both EPD and LPD were most significantly changed between the HC and TBI/stroke groups in parietal and temporal hotspots, most probably due the higher SNR in the hotspots that are proximal to the stimulation area or contralateral to it. LPD was significantly changed in the stroke group alone, whereas EPD was significantly different in TBI alone. This may originate from the differences in age groups, as the TBI group and control are significantly younger, or might possibly arise from different natures of injuries that for stroke might be more focal and for TBI more diffused. These differences should be further explored and may potentially provide additional insight. The fact that WFA alone was significantly affected in both the TBI and stroke groups for all recording hotspots may indicate the notion that different injuries (location, sizes, etc.) are represented in different phases or latencies (late/early) of the TEP response and therefore were not as significantly noticeable in the EPD and LPD but rather in the WFA parameter that represents the entire response pattern capturing all possible changed parameters of the signal. DTI analysis demonstrated that, as expected, WM integrity was significantly compromised in both the TBI and stroke groups compared with HC in most ROIs. This is most probably due to the variability in injury locations and sizes. A multidimensional approach utilizing linear SVM model, which incorporates all DELPHI output measures, displays high classification performance of healthy vs. TBI or stroke populations with a balanced accuracy of 0.81 ± 0.02 and AUC of 0.88 ± 0.01. Moreover, a multivariate linear regression model revealed high correlation between DELPHI output measures and various WM fibers, which is consistent with the decrease of FA values in a wide range of ROIs in the stroke and TBI groups, compared with HC. Interestingly, higher sensitivity to WM integrity was observed in fibers that are in closest proximity to TMS stimulation site (M1L), namely, the highest values were recorded in the frontal and left fibers, which are proximal to the TMS stimulation site. These data support DELPHI (TMS-EEG) as a tool for neurophysiological evaluation of network connectivity and its potential as an affordable, safe and available tool for monitoring and evaluating brain network structural and functional abnormalities in specific neuronal circuits. These results are supported by previous studies (Manganotti et al., 2015; Lanza et al., 2017; Hordacre et al., 2019) and indicate that in order to get a more comprehensive and localized evaluation of functional and structural brain connectivity, integrity, and plasticity, at least two contralateral stimulation sites are required. Moreover, direct TMS stimulation of frontal, medial, and posterior areas may increase TMS-EEG sensitivity to both structural and functional ROIs. TMS-EEG technology has been shown to provide insight into the evaluation and monitoring of functional effective connectivity in various brain disorders, such as Alzheimer’s disease (AD), stroke, and TBI prognosis and rehabilitation prediction (Bortoletto et al., 2015; Manganotti et al., 2015; Tremblay et al., 2019). Our study supports the clinical utility of TMS-EEG in brain disorders of functional and structural effective connectivity, displays high sensitivity of DELPHI neurophysiological measures to WM-related structural connectivity changes as measured with DTI, and supports specific fiber localization correlated to the stimulation area. It is important to note that the current study classifiers did not consider age-related WM and GM changes, such as minor lacunar infracts, lesions, and brain atrophy, that may affect “brain health” status in the HC group; however, these measures were addressed in the regression model with FA analysis of WM fibers for all study groups. Further studies are required to address the functional sensitivity of changes in neuronal circuits and explore the potential predictive value of this tool for the early detection, predictive recovery, and prognosis of WM-related brain abnormalities and diseases. Future studies should include populations suffering from brain abnormalities that may or may not be detected by conventional clinical MRI/CT scans in order to establish detection of subtle WM changes in clinical conditions, such as mild TBI, cerebral small vessels disease (CSVD), etc., and record clinical measures of cognitive and physiological performance. The current study supports previous studies that display TEP as a biomarker for brain excitability and functional connectivity (Bagattini et al., 2019; Opie et al., 2019; Rossini et al., 2020). Here, we provide further evidence for the physiological relevance of probing specific networks (such as the motor network that was stimulated in the current study) with TMS-EEG technology in order to record structural or functional WM abnormalities in brain injuries or other diseases. These results demonstrate the potential clinical value of DELPHI output measures in differentiating brain-related diseases and abnormalities, providing a reliable, safe and easy-to-use tool for monitoring WM-related connectivity changes associated with brain-related abnormalities, such as brain injuries and degeneration.

## Conclusion

The ability to facilitate between clinical observations, structural and functional brain connectivity, and network physiology is crucial in order to achieve an optimal clinical care. The data presented in this study support the concept that TMS-EEG physiological measures provide specific accessible insight into the human connectome that may help to narrow the gap between anatomical data, effective network function, and clinical observations, thus, providing a clinical tool for monitoring brain network function and brain health.

The DELPHI automated acquisition and analysis system can be used in order to monitor brain health throughout aging and may enable the early detection of abnormal pathophysiological changes leading to neurodegeneration.

## Data Availability Statement

The original contributions presented in the study are included in the article/supplementary materials, further inquiries can be directed to the corresponding author/s.

## Ethics Statement

The studies involving human participants were reviewed and approved by Shamir Medical center review board. The patients/participants provided their written informed consent to participate in this study.

## Author Contributions

NZ with OL-L, HF, and ID have determined the study design and criteria. NZ conducted most of the laboratory works. OL-L and HF designed most of the DELPHI software algorithm. HF and ID contributed in designing and writing most of the manuscript and submitting the manuscript for publication. SE was principal investigator in the study. ES was responsible for the MRI imaging analysis in the study. DH, DT, SE, and ES contributed in assuring the methods and the quality of the results and with reviewing the manuscript and approving for publication of the content. All authors contributed to the article and approved the submitted version.

## Conflict of Interest

OL-L, NZ, DH, DT, HF, and ID are employed by QuantalX neuroscience. The remaining authors declare that the research was conducted in the absence of any commercial or financial relationships that could be construed as a potential conflict of interest.
